# Author Correction: Multi-ancestry meta-analysis of genome-wide association studies discovers 67 new loci associated with chronic back pain

**DOI:** 10.1038/s41467-025-57504-3

**Published:** 2025-03-03

**Authors:** Ian B. Stanaway, Pradeep Suri, Niloofar Afari, Daniel Dochtermann, Armand Gerstenberger, Saiju Pyarajan, Eric J. Roseen, Million Veteran Program, Ian B. Stanaway, Pradeep Suri, Daniel Dochtermann, Armand Gerstenberger, Saiju Pyarajan, Marianna Gasperi, Marianna Gasperi

**Affiliations:** 1 VA Puget Sound Health Care System (VAPSHCS), Seattle, WA USA; 2 Department of Nephrology, University of Washington, Seattle, WA USA; 3 Seattle Epidemiologic Research and Information Center, VAPSCHS, Seattle, WA USA; 4 Department of Rehabilitation Medicine, University of Washington, Seattle, WA USA; 5 Clinical Learning, Evidence, and Research (CLEAR) Center, University of Washington, Seattle, WA USA; 6 Department of Psychiatry, University of California San Diego, La Jolla, CA USA; 7 VA San Diego Healthcare System (VASDHS), San Diego, CA USA; 8 Center of Excellence for Stress and Mental Health, VASDHS, San Diego, CA USA; 9 Center for Data and Computational Sciences (C-DACS), VA Boston Healthcare System (VABHS), Boston, MA USA; 10 Mental Illness Research Education and Clinical Center (MIRECC), VAPSHCS, Seattle, WA USA; 11 Section of General Internal Medicine, Department of Medicine, Boston University Chobanian & Avedision School of Medicine and Boston Medical Center, Boston, MA USA; 12 Department of Physical Medicine & Rehabilitation, VA Boston Healthcare System, Boston, MA USA; 13 Department of Psychiatry and Behavioral Sciences, University of Washington, Seattle, WA USA

**Keywords:** Diseases, Genome-wide association studies, Chronic pain

Correction to: *Nature Communications* 10.1038/s41467-024-55326-3, published online 11 February 2025

In this article the author name Eric J. Roseen was incorrectly written as Eric J. Rosen. The original article has been corrected.

