# Supplementary material for: Multi-ancestry meta-analysis of genome-wide association studies discovers 67 new loci associated with chronic back pain
Source: Nat Commun. 2025 Feb 11;16:1525. doi: 10.1038/s41467-024-55326-3 (PMC11814113; doi:10.1038/s41467-024-55326-3)
Supplement: Supplementary file 1 — Supplementary Information [file 41467_2024_55326_MOESM1_ESM.pdf]

Figure S1. GWAS Manhattan plot of EHR defined CBP in European Ancestry (EUR) Participants

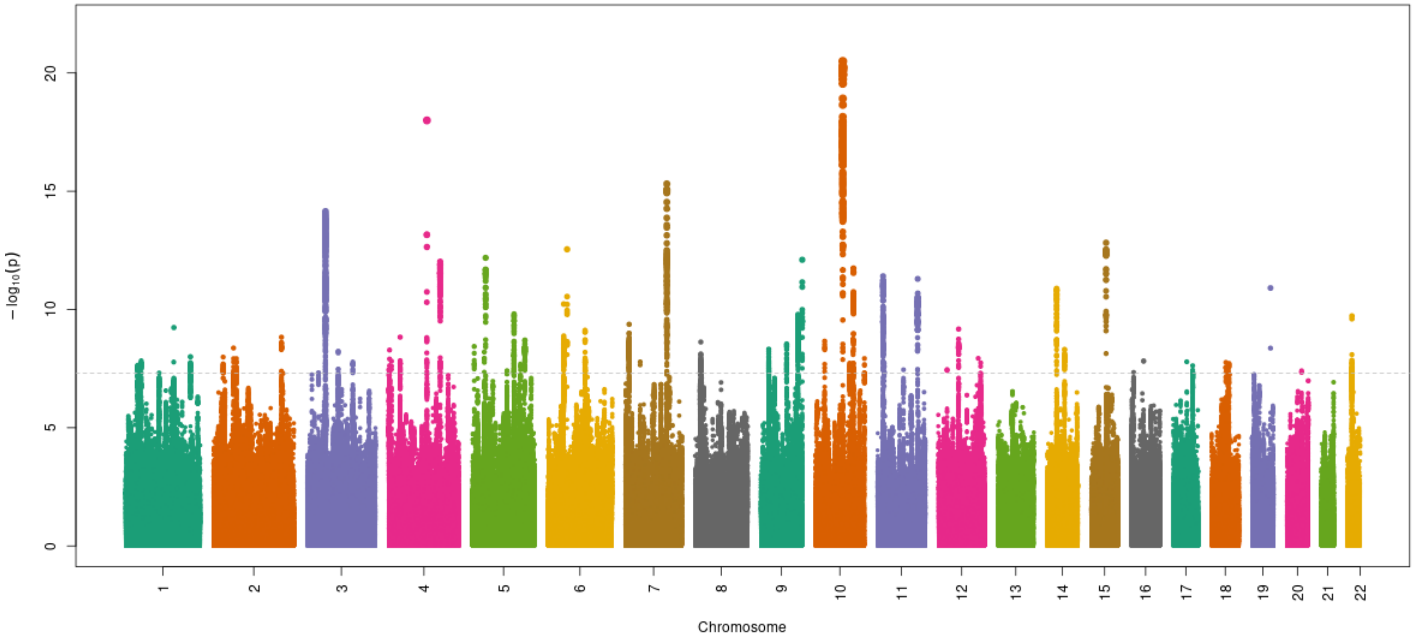

Figure S2. GWAS Manhattan plot of EHR defined CBP in African Ancestry (AFR) Participants

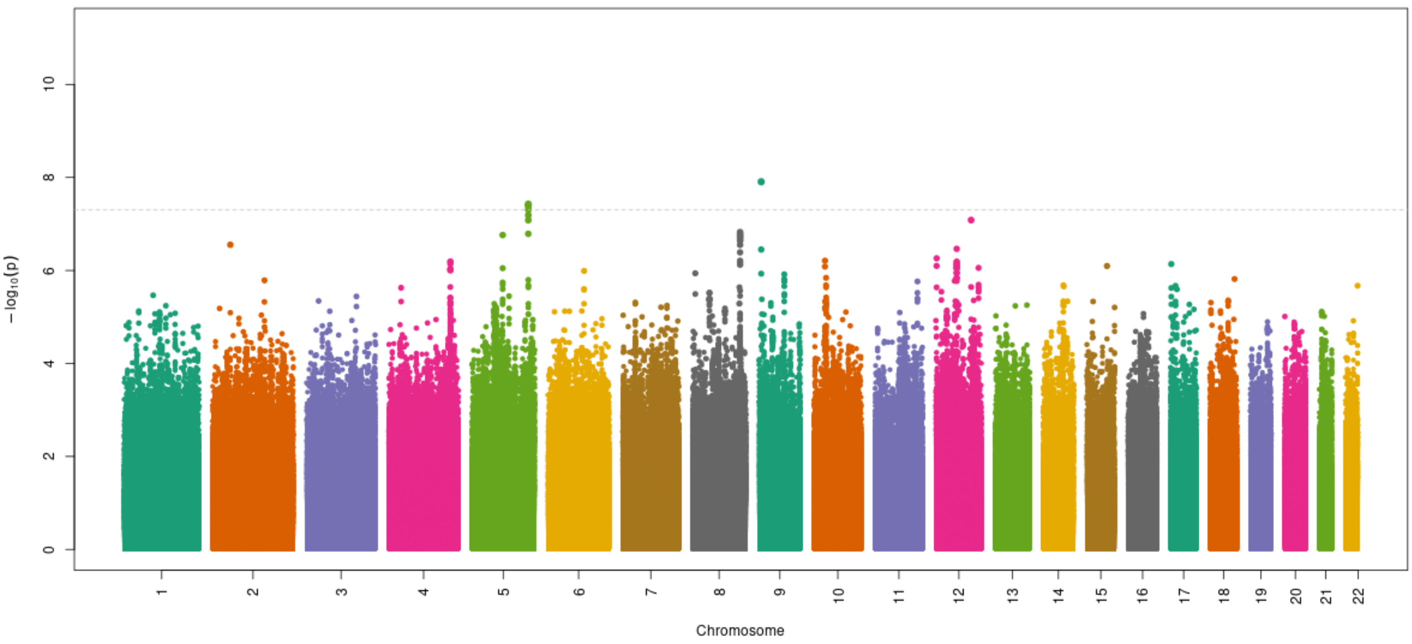

Figure S3. GWAS Manhattan plot of EHR defined CBP in Hispanic Ethnicity (HIS) Participants

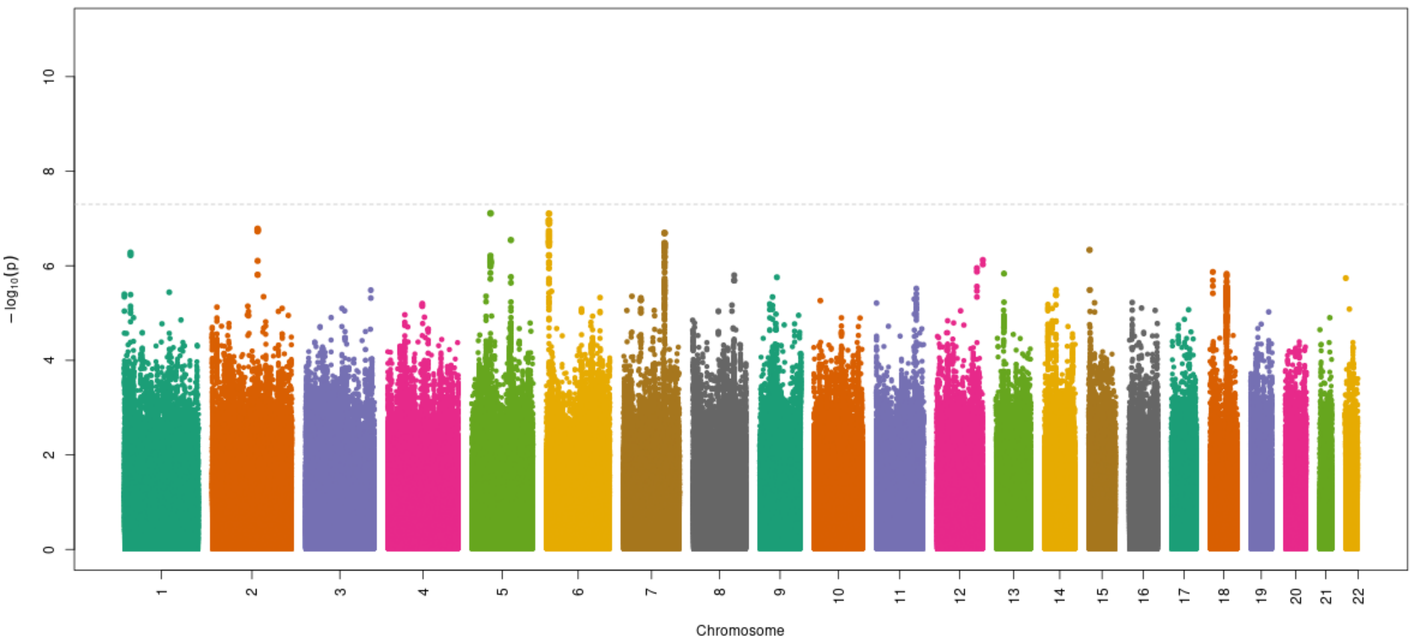

Figure S4. Multi-Ancestry Meta-Analysis GWAS QQ plot of EHR defined CBP

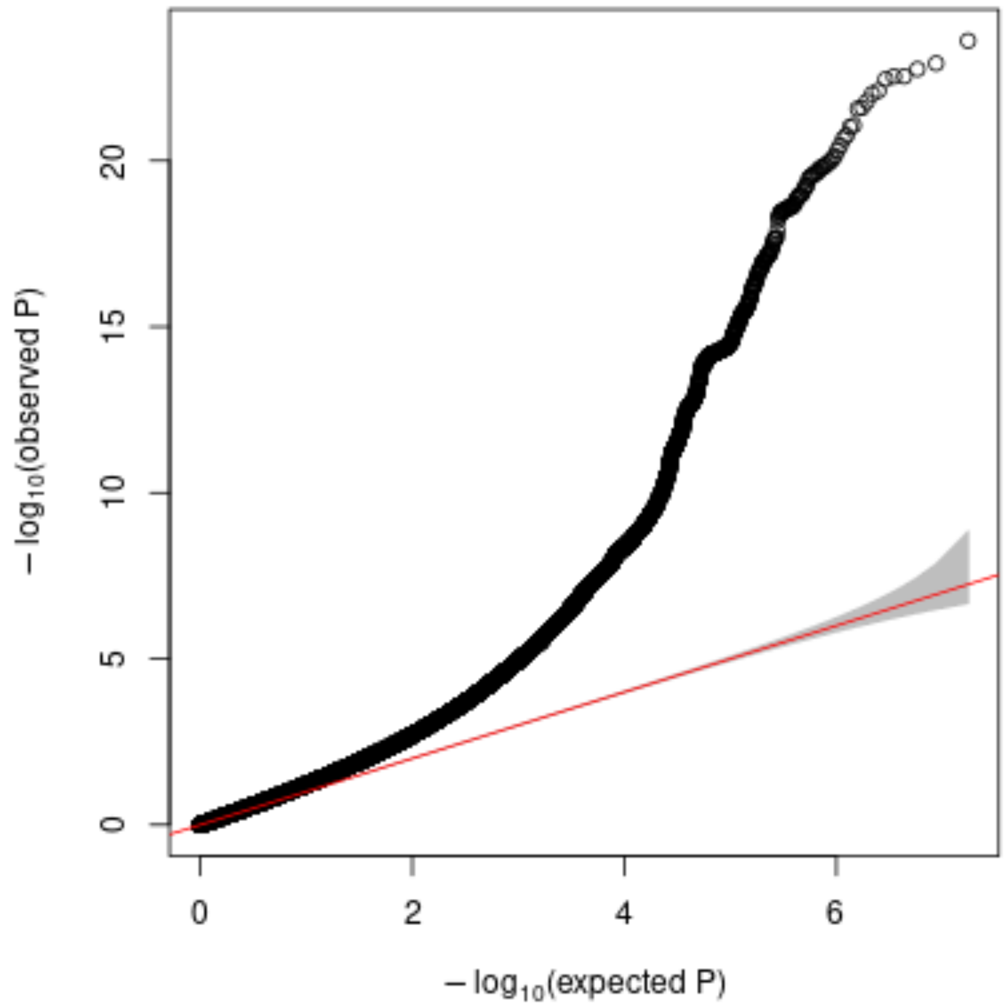

Figure S5. GWAS QQ plot of EHR defined CBP in European Ancestry (EUR) Participants

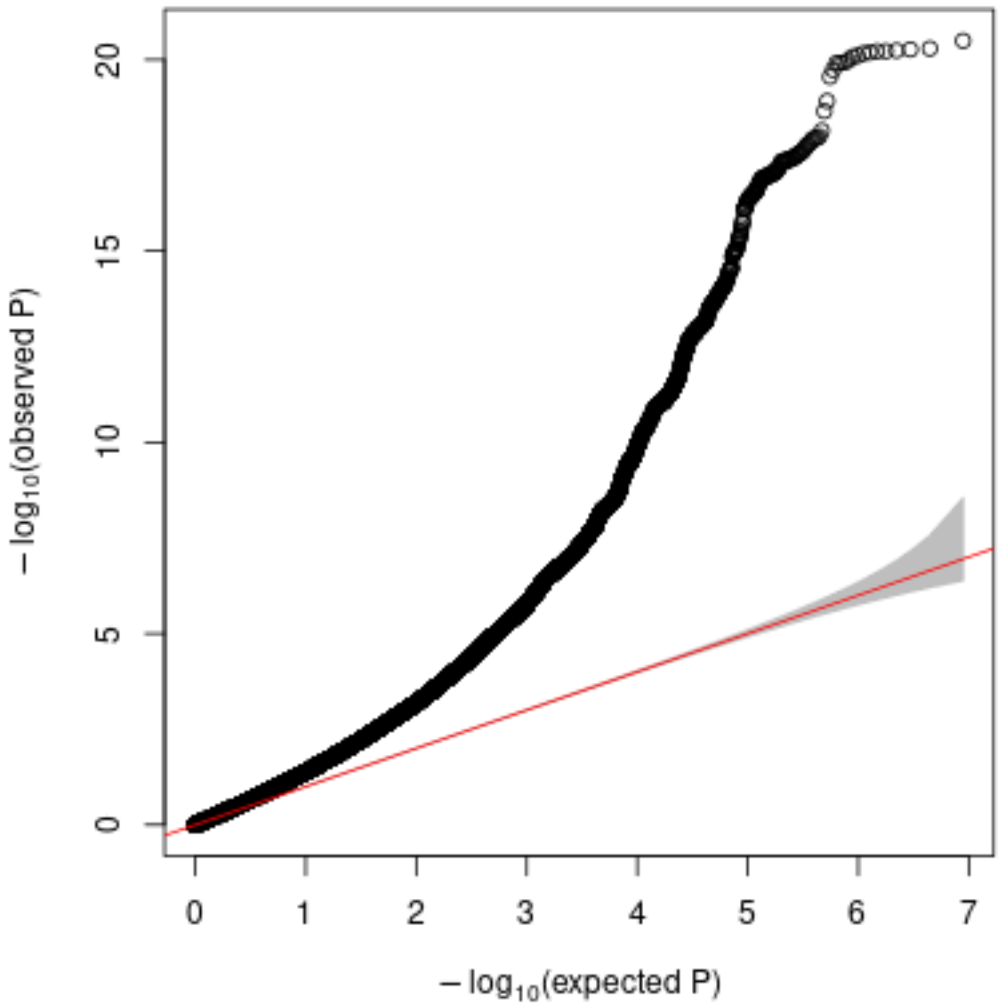

Figure S6. GWAS QQ plot of EHR defined CBP in African Ancestry (AFR) Participants

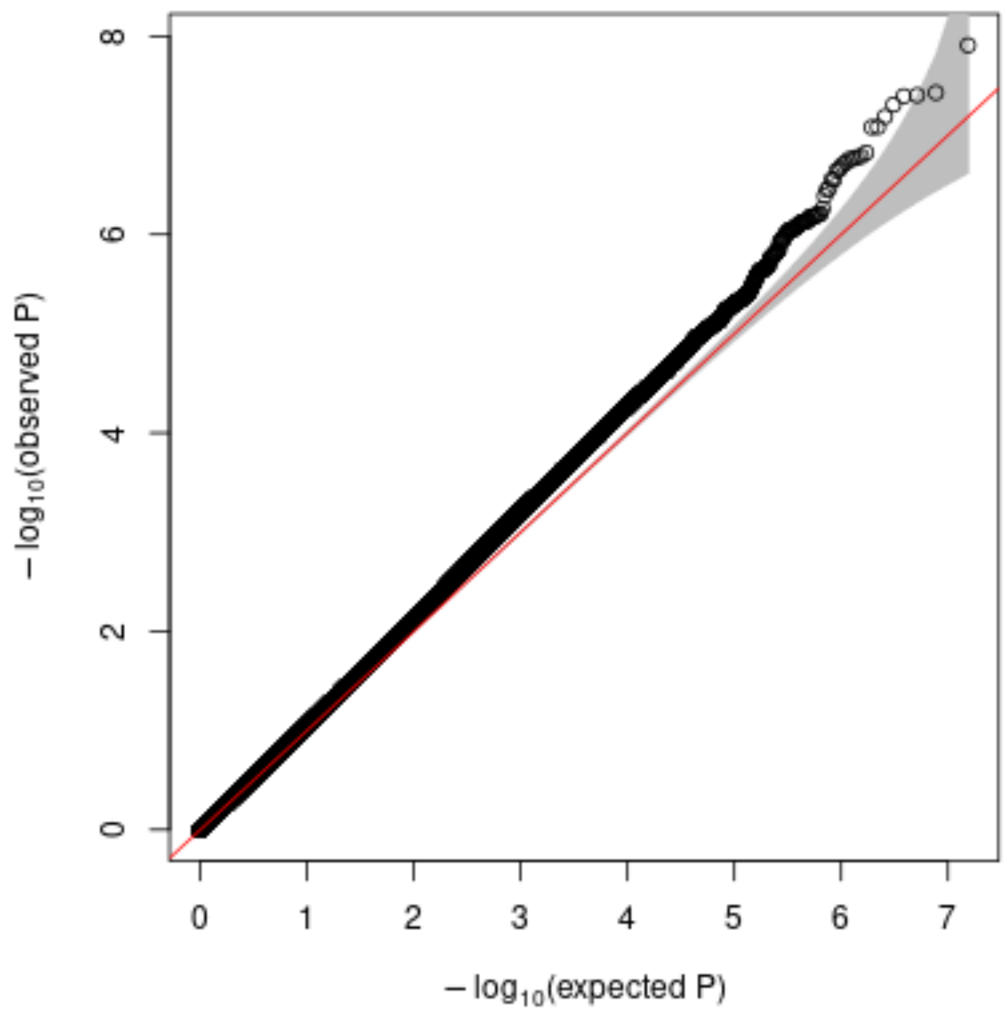

Figure S7. GWAS QQ plot of EHR defined CBP in Hispanic Ethnicity (HIS) Participants

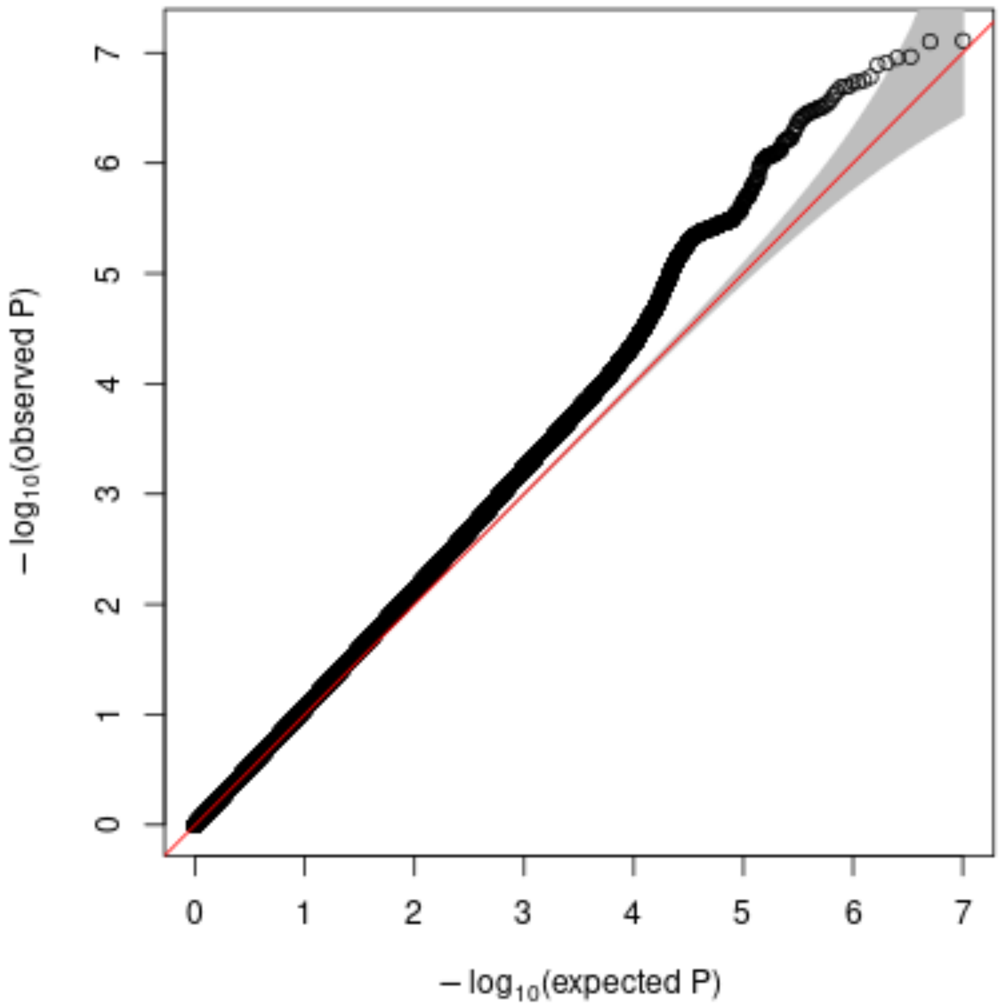

Figure S8. MiXeR genetic overlap between MVP (A) EUR and AFR HARE-defined ancestry cohorts (B) EUR and HIS HARE-defined ancestry cohorts, and (C) HIS and AFR HARE-defined ancestry cohorts. Venn diagrams report numbers in thousands of variant risk loci (standard error) related to the first trait (blue circle), second trait (orange circle), and the mutually shared variants (grey overlap). Genetic correlation is depicted as a number and red progress bar. Conditional QQ plot of trait 1 on subset 2 and trait 2 on subset 1 in the two center plots of each sub-figure. The model log likelihoods based on the number of causal variants are shown in the rightmost plots. The solid blue line reflects the average likelihood across the 20 MiXeR runs. Dotted blue lines reflect likelihoods of individual runs.

(A)

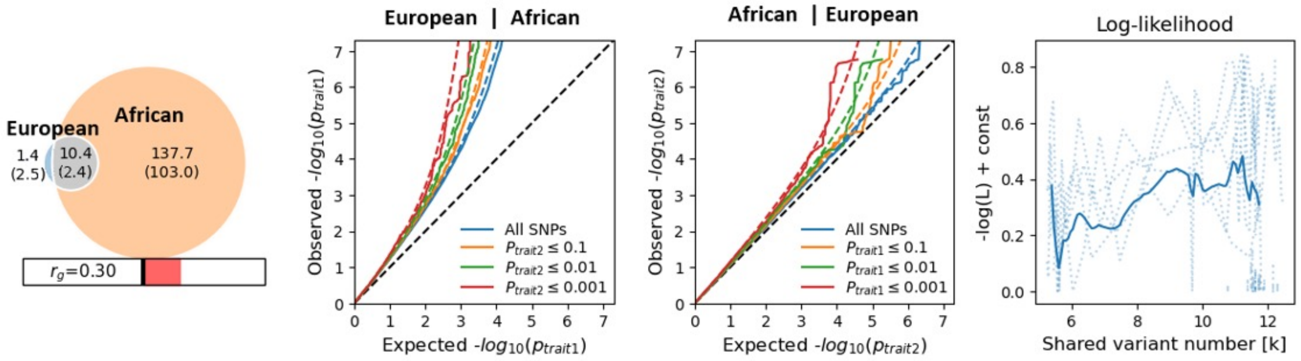

(B)

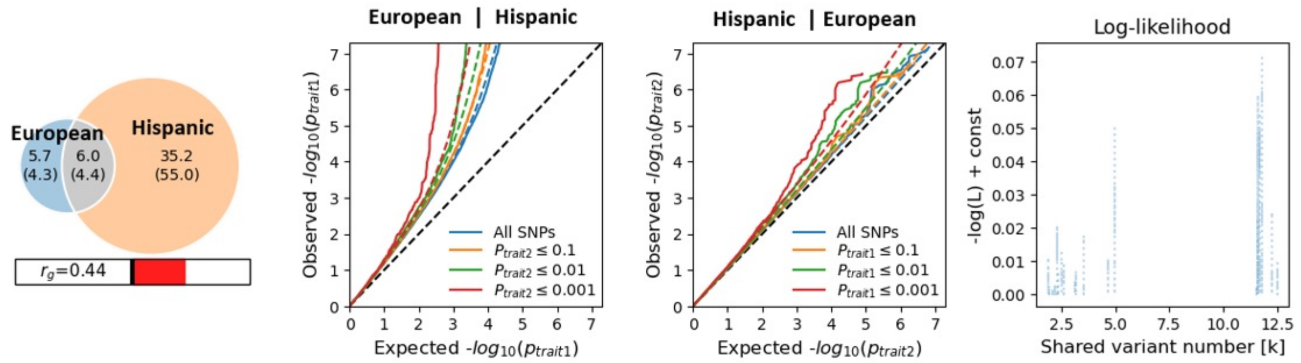

(C)

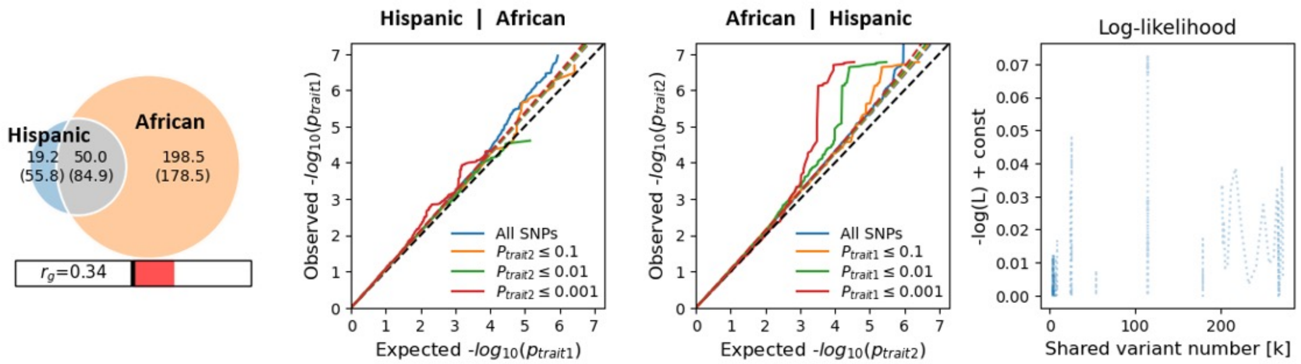

Figure S9. (A) FUMA multi-ancestry analysis MAGMA GTEx functional tissue enrichment histograms of tissue-specific localization of QTLs

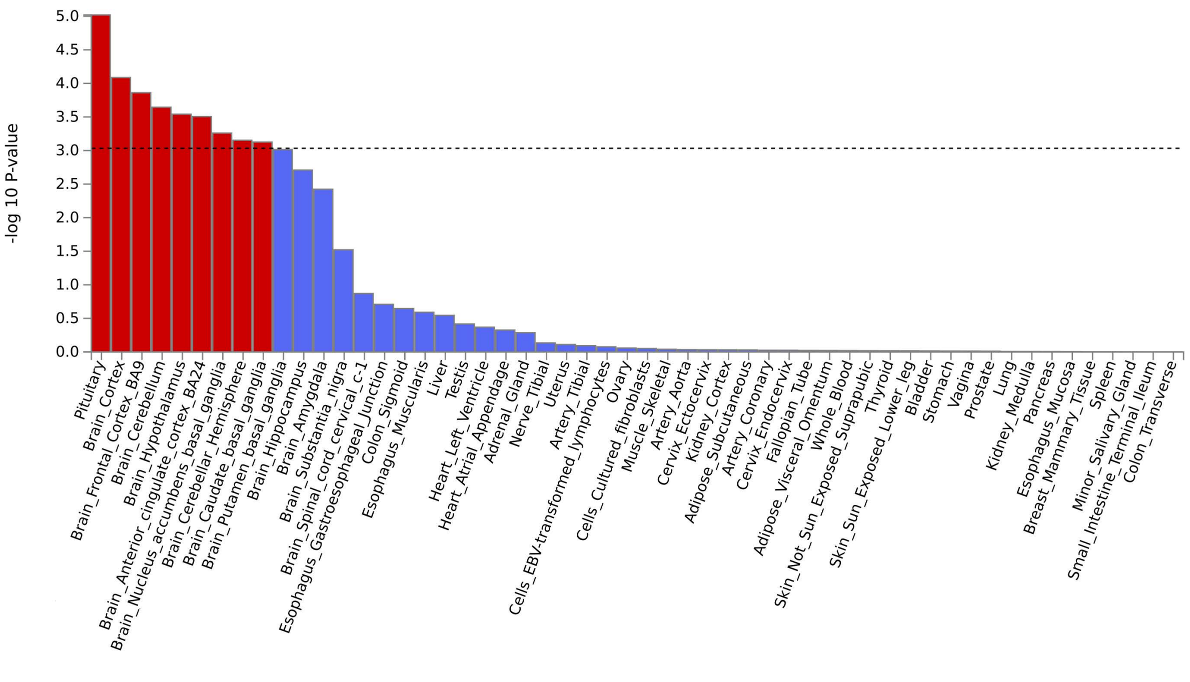

Figure S9 (B) FUMA multi-ancestry analysis MAGMA GTEx functional tissue enrichment histograms grouped by tissue context

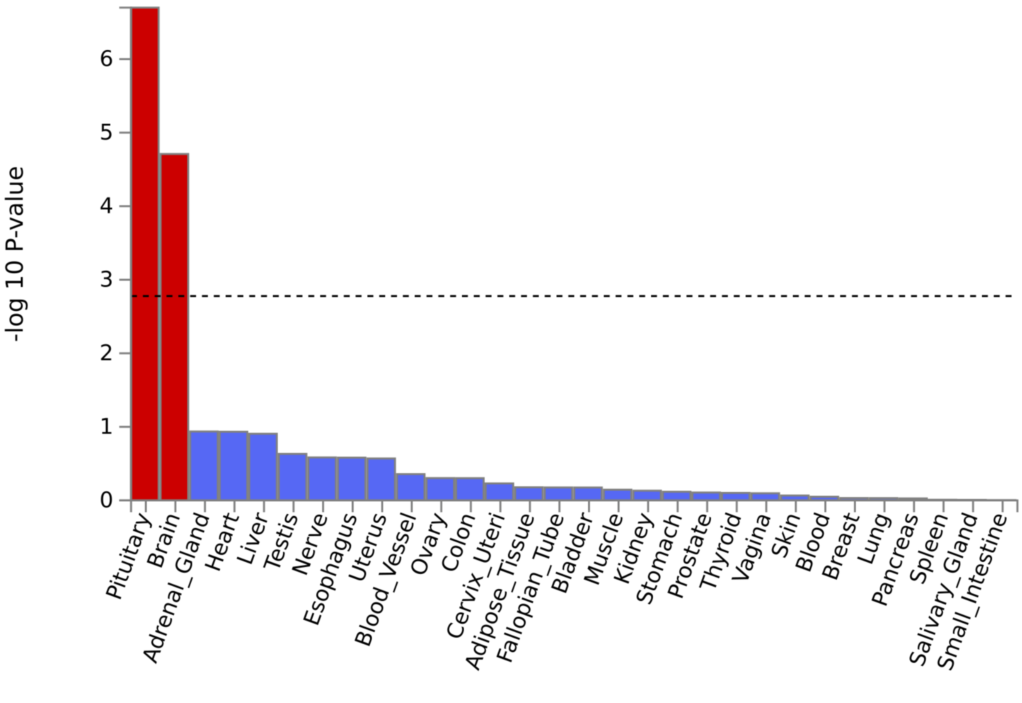

Figure S10. Description: Cell type analyses conducted within the FUMA Cell Type module using the GSE67835_Human_Coretex_woFetal dataset MAGMA cell specificity analyses. A) Per-data cell type specificity; B) Significant cell types across datasets (Step 1); C) Independent cell type associations based on within-dataset conditional analyses; D) Pair-wise cross-datasets conditional analyses.

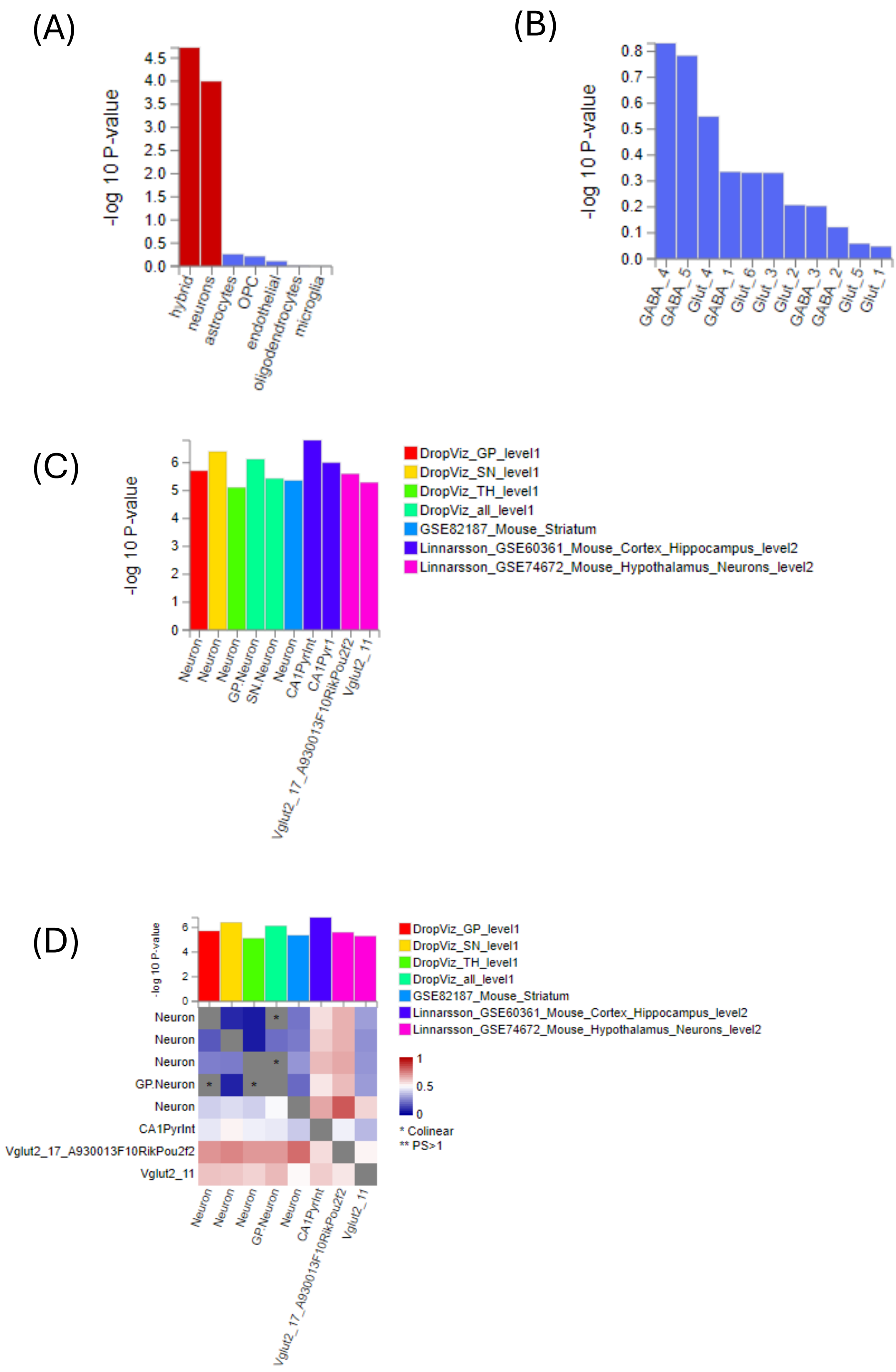

VA Million Veteran Program
Core Acknowledgment for Publication

MVP Program Office

- Sumitra Muralidhar, Ph.D., Program Director
  US Department of Veterans Affairs, 810 Vermont Avenue NW, Washington, DC 20420
- Jennifer Moser, Ph.D., Associate Director, Scientific Programs
  US Department of Veterans Affairs, 810 Vermont Avenue NW, Washington, DC 20420
- Jennifer E. Deen, B.S., Associate Director, Cohort & Public Relations
  US Department of Veterans Affairs, 810 Vermont Avenue NW, Washington, DC 20420

MVP Executive Committee

- Co-Chair: Philip S. Tsao, Ph.D.
  VA Palo Alto Health Care System, 3801 Miranda Avenue, Palo Alto, CA 94304
- Co-Chair: Sumitra Muralidhar, Ph.D.
  US Department of Veterans Affairs, 810 Vermont Avenue NW, Washington, DC 20420
- J. Michael Gaziano, M.D., M.P.H.
  VA Boston Healthcare System, 150 S. Huntington Avenue, Boston, MA 02130
- Elizabeth Hauser, Ph.D.
  Durham VA Medical Center, 508 Fulton Street, Durham, NC 27705
- Amy Kilbourne, Ph.D., M.P.H.
  VA HSR&D, 2215 Fuller Road, Ann Arbor, MI 48105
- Michael Matheny, M.D., M.S., M.P.H.
  VA Tennessee Valley Healthcare System, 1310 24th Ave. South, Nashville, TN 37212
- Dave Oslin, M.D.
  Philadelphia VA Medical Center, 3900 Woodland Avenue, Philadelphia, PA 19104
- Deepak Voora, MD
  Durham VA Medical Center, 508 Fulton Street, Durham, NC 27705

MVP Co-Principal Investigators

- J. Michael Gaziano, M.D., M.P.H.
  VA Boston Healthcare System, 150 S. Huntington Avenue, Boston, MA 02130
- Philip S. Tsao, Ph.D.
  VA Palo Alto Health Care System, 3801 Miranda Avenue, Palo Alto, CA 94304

MVP Core Operations

- Jessica V. Brewer, M.P.H., Director, MVP Cohort Operations
  VA Boston Healthcare System, 150 S. Huntington Avenue, Boston, MA 02130
- Mary T. Brophy M.D., M.P.H., Director, VA Central Biorepository
  VA Boston Healthcare System, 150 S. Huntington Avenue, Boston, MA 02130
- Kelly Cho, M.P.H, Ph.D., Director, MVP Phenomics
  VA Boston Healthcare System, 150 S. Huntington Avenue, Boston, MA 02130

- Lori Churby, B.S., Director, MVP Regulatory Affairs
  VA Palo Alto Health Care System, 3801 Miranda Avenue, Palo Alto, CA 94304
- Scott L. DuVall, Ph.D., Director, VA Informatics and Computing Infrastructure (VINCI)
  VA Salt Lake City Health Care System, 500 Foothill Drive, Salt Lake City, UT 84148
- Saiju Pyarajan Ph.D., Director, Data and Computational Sciences
  VA Boston Healthcare System, 150 S. Huntington Avenue, Boston, MA 02130
- Robert Ringer, Pharm.D., Director, VA Albuquerque Central Biorepository
  New Mexico VA Health Care System, 1501 San Pedro Drive SE, Albuquerque, NM 87108
- Luis E. Selva, Ph.D., Director, MVP Biorepository Coordination
  VA Boston Healthcare System, 150 S. Huntington Avenue, Boston, MA 02130
- Shahpoor (Alex) Shayan, M.S., Director, MVP PRE Informatics
  VA Boston Healthcare System, 150 S. Huntington Avenue, Boston, MA 02130
- Brady Stephens, M.S., Principal Investigator, MVP Information Center
  Canandaigua VA Medical Center, 400 Fort Hill Avenue, Canandaigua, NY 14424
- Stacey B. Whitbourne, Ph.D., Director, MVP Cohort Development and Management
  VA Boston Healthcare System, 150 S. Huntington Avenue, Boston, MA 02130

MVP Publications and Presentations Committee

- Co-Chair: Themistocles L. Assimes, M.D., Ph. D
  VA Palo Alto Health Care System, 3801 Miranda Avenue, Palo Alto, CA 94304
- Co-Chair: Adriana Hung, M.D.; M.P.H
  VA Tennessee Valley Healthcare System, 1310 24th Ave. South, Nashville, TN 37212
- Co-Chair: Henry Kranzler, M.D.
  Philadelphia VA Medical Center, 3900 Woodland Avenue, Philadelphia, PA 19104
