# Supplementary material for: Multi-ancestry meta-analysis of genome-wide association studies discovers 67 new loci associated with chronic back pain
Source: Nat Commun. 2025 Feb 11;16:1525. doi: 10.1038/s41467-024-55326-3 (PMC11814113; doi:10.1038/s41467-024-55326-3)
Supplement: Supplementary file 2 — Description of Additional Supplementary Files [file 41467_2024_55326_MOESM2_ESM.docx]

**Description of Additional Supplementary Files**

**File name: Supplementary Data 1**

Description: Extra Demographic Information

**File name: Supplementary Data 2**

Description: Genome-wide significant variants in multi-ancestry analysis

**File name: Supplementary Data 3**

Description: Genome-wide significant variants in European ancestry analysis

**File name: Supplementary Data 4**

Description: Genome-wide significant variants in African ancestry analysis

**File name: Supplementary Data 5**

Description: Results for Linkage Disequilibrium Score Regression (LDSR) of heritability

**File name: Supplementary Data 6**

Description: Summary statistics from Bjornsdottir et al. used in Bonferroni corrected replication of MVP-CBP GWAS significant variants in the multi-ancestry meta-analysis

**File name: Supplementary Data 7**

Description: Summary statistics from Bjornsdottir et al., which nominally replicate MVP-CBP GWAS significant variants in the multi-ancestry meta-analysis

**File name: Supplementary Data 8**

Description: Summary statistics from Bjornsdottir et al. used in Bonferroni corrected replication of MVP-CBP GWAS significant variants in the European ancestry analysis

**File name: Supplementary Data 9**

Description: Summary statistics from Bjornsdottir et al., which nominally replicate MVP-CBP GWAS significant variants in the European ancestry analysis

**File name: Supplementary Data 10**

Description: Summary statistics from Bjornsdottir et al., which nominally replicate MVP-CBP GWAS significant variants in the African ancestry analysis

**File name: Supplementary Data 11**

Description: Prior back pain GWASs' lead variants annotated for PMID and genes

**File name: Supplementary Data 12**

Description: Prior back pain GWASs' lead variants annotated by the results from the multi-ancestry meta-analysis of MVP-CBP

**File name: Supplementary Data 13**

Description: Annotation of the novel versus known loci from prior publications of closely related back pain phenotypes

**File name: Supplementary Data 14**

Description: Results for Linkage Disequilibrium Score Regression (LDSR) of genetic correlations between each ancestry stratas' summary statistics

**File name: Supplementary Data 15**

Description: Results for Linkage Disequilibrium Score Regression (LDSR) of genetic correlations between different back pain and similar pain outcomes GWAS summary statistics

**File name: Supplementary Data 16**

Description: Results for Linkage Disequilibrium Score Regression (LDSR) of genetic correlations between different traits GWAS summary statistics provided at <https://vl.genoma.io/>

**File name: Supplementary Data 17**

Description: FUMA identification of the lead variants at each locus of the multi-ancestry meta-analysis

**File name: Supplementary Data 18**

Description: FUMA magma GTEx general tissue analysis output

**File name: Supplementary Data 19**

Description: FUMA magma GTEx-specific tissue analysis output

**File name: Supplementary Data 20**

Description: Result of the Mendelian Randomization (MR) of MVP-CBP significant variants with GTEx summary statistics used as instrument variables applied to the outcome of "Dorsalgia" spinal pain from Bjornsdottir et al.

**File name: Supplementary Data 21**

Description: FUMA identification of the lead variants at each locus of the European ancestry analysis

**File name: Supplementary Data 22**

Description: FUMA identification of the lead variants at each locus of the African ancestry analysis

**File name: Supplementary Data 23**

Description: FUMA identification of the genes at each locus of the multi-ancestry meta-analysis

**File name: Supplementary Data 24**

Description: FUMA identification of the genes at each locus of the European ancestry analysis

**File name: Supplementary Data 25**

Description: FUMA identification of the genes at each locus of the African ancestry analysis
