# Supplementary material for: Multi-ancestry meta-analysis of genome-wide association studies discovers 67 new loci associated with chronic back pain
Source: Nat Commun. 2025 Feb 11;16:1525. doi: 10.1038/s41467-024-55326-3 (PMC11814113; doi:10.1038/s41467-024-55326-3)
Supplement: Supplementary file 4 — Reporting Summary [file 41467_2024_55326_MOESM4_ESM.pdf]

nature portfolio

Corresponding author(s): Marianna Gasperi, Ph.D.

Last updated by author(s): 6/2924

Reporting Summary

Nature Portfolio wishes to improve the reproducibility of the work that we publish. This form provides structure for consistency and transparency in reporting. For further information on Nature Portfolio policies, see our Editorial Policies and the Editorial Policy Checklist.

Statistics

For all statistical analyses, confirm that the following items are present in the figure legend, table legend, main text, or Methods section.

n/a	Confirmed	
☐	☒	The exact sample size (n) for each experimental group/condition, given as a discrete number and unit of measurement
☐	☒	A statement on whether measurements were taken from distinct samples or whether the same sample was measured repeatedly
☐	☒	The statistical test(s) used AND whether they are one- or two-sided<br>*Only common tests should be described solely by name; describe more complex techniques in the Methods section.*
☐	☒	A description of all covariates tested
☐	☒	A description of any assumptions or corrections, such as tests of normality and adjustment for multiple comparisons
☐	☒	A full description of the statistical parameters including central tendency (e.g. means) or other basic estimates (e.g. regression coefficient) AND variation (e.g. standard deviation) or associated estimates of uncertainty (e.g. confidence intervals)
☐	☒	For null hypothesis testing, the test statistic (e.g. F, t, r) with confidence intervals, effect sizes, degrees of freedom and P value noted<br>*Give P values as exact values whenever suitable.*
☒	☐	For Bayesian analysis, information on the choice of priors and Markov chain Monte Carlo settings
☒	☐	For hierarchical and complex designs, identification of the appropriate level for tests and full reporting of outcomes
☐	☒	Estimates of effect sizes (e.g. Cohen's d, Pearson's r), indicating how they were calculated

Our web collection on statistics for biologists contains articles on many of the points above.

Software and code

Policy information about availability of computer code

Data collection	No specific software was used for data collection in this study
Data analysis	MVP genotype data imputation was performed with Minimac4 using TopMed reference panel data. Principal components were calculated for each ancestry using PLINK 2.0 alpha18 on genotyped data. Harmonizing Genetic Ancestry and Self-identified Race/Ethnicity groups were used to define race/ethnicity. GWAS analysis was conducted for the three ancestry groups using logistic regression to test the association between CBP and imputed dosages using REGENIE v2.2.26. Meta-analysis was conducted across three ancestries (EUR, AFR, HIS; n=553,601) using the METAL software. Functional Mapping and Annotation of Genome-Wide Association Studies (FUMA) was used to annotate variant results. The MAGMA v1.06 method and the MsigDB v5.2 database from FUMA were used. Linkage disequilibrium score regression (LDSC) was used to estimate the SNP-based heritability of CBP-MVP for each ancestry. LDSC regression was used to assess the genetic correlation of CBP-MVP between each ancestry stratum in MVP. Cross-trait LDSC regression was performed on various conditions the Complex Trait Genetics Virtual Lab (https://vl.genoma.io/). We conducted post hoc Mendelian randomization (MR) analyses using the TwoSampleMR R package (v0.5.6). We used the mixed effects score regression (MiXeR) framework to estimate the genetic overlap between three ancestry strata of MVP-CBP, the degree of polygenicity, and the current power to detect causal loci.

For manuscripts utilizing custom algorithms or software that are central to the research but not yet described in published literature, software must be made available to editors and reviewers. We strongly encourage code deposition in a community repository (e.g. GitHub). See the Nature Portfolio guidelines for submitting code & software for further information.

Data

Policy information about availability of data

All manuscripts must include a data availability statement. This statement should provide the following information, where applicable:

- Accession codes, unique identifiers, or web links for publicly available datasets
- A description of any restrictions on data availability
- For clinical datasets or third party data, please ensure that the statement adheres to our policy

Raw MVP data are protected and are not available due to privacy reasons. GWAS and meta-analysis summary statistics will be available in dbGaP (https://www.ncbi.nlm.nih.gov/gap/) upon publication under accession phs001672. MVP summary data access can be obtained by submitting a data access request through dbGaP.

Research involving human participants, their data, or biological material

Policy information about studies with human participants or human data. See also policy information about sex, gender (identity/presentation), and sexual orientation and race, ethnicity and racism.

Reporting on sex and gender	Gender was determined from self report. The MVP sample was approximately 9% (N=504,527 men, 49,074 women). The joint analysis includes both men and women for EUR, HIS, and AFR ancestry strata and EUR men and EUR women samples separately. HIS and AFR men and women were not evaluated separately due to sample size limitations. The total number of participants by gender is reported in the manuscript. Analyses for EUR men and EUR women were carried out and are reported in the results.
Reporting on race, ethnicity, or other socially relevant groupings	The study design was specifically tailored to consider the crucial factors of race, ethnicity, and ancestry. These factors play a significant role in understanding the genetic variations and their implications, making them essential components of our research.<br>Harmonizing Genetic Ancestry and Self-identified Race/Ethnicity (HARE) groups were used to define race/ethnicity.19 Briefly, HARE enhances classification by integrating self-identified race/ethnicity (SIRE) and genetically inferred ancestry (GIA). HARE ensures accurate classification by using GIA to refine and, if necessary, impute SIRE, improving the reliability of race/ethnicity assignment in genetic research.<br>553,601 participants had available phenotype and genotype information and were used for GWAS analysis (Table 1). The HARE sample sizes for European ancestry (EUR, n=402,005 total; 201,443 cases, 200,562 controls), African ancestry (AFR, n=106,465 total; 66,366 cases, 40,099 controls), and Hispanic ethnicity (HIS, n=45,131 total; 26,914 cases, 18,217 controls) groups were used in this analysis. People of East Asian and South Asian ancestry were not analyzed due to the low numbers of Asian individuals in MVP.
Population characteristics	The average age of participants was 61.7 years old. MVP samples were genotyped using a 723,305 single nucleotide polymorphism (SNP) Affymetrix Axiom Biobank array. With 294,723 cases and 258,878 controls from three HARE ancestral backgrounds, the sample had an overall CBP phenotypic prevalence of 53.2%.
Recruitment	With the 2022 MVP data release, 819,417 participants were enrolled in the MVP, and 662,681 had genetic data available for analyses reported here. MVP data include self-reported survey, electronic health record (EHR), and genetic data.
Ethics oversight	The VA cIRB and the Research and Development Committee at VA San Diego Healthcare System and VA Puget Sound System approved the current analyses. Details on the cohorts and phenotypes are provided in the supplement.

Note that full information on the approval of the study protocol must also be provided in the manuscript.

Field-specific reporting

Please select the one below that is the best fit for your research. If you are not sure, read the appropriate sections before making your selection.

☒ Life sciences ☐ Behavioural & social sciences ☐ Ecological, evolutionary & environmental sciences

For a reference copy of the document with all sections, see nature.com/documents/nr-reporting-summary-flat.pdf

Life sciences study design

All studies must disclose on these points even when the disclosure is negative.

Sample size	Sample size was not predetermined but reflects the results of the phenotyping effort of individuals with CBP in MVP with genetic data.
Data exclusions	Data exclusions were performed based on either failure of predetermined data quality control or planned phenotype exclusions to ensure the validity of case/control criteria. Individuals observed to have low genotyping quality were excluded. Ancestries other than European, African, Hispanic/LatinX were excluded due to insufficient sample size for meaningful analysis in the currently available data. For phenotype-based exclusions, individuals with insufficient EHR data, missing fender, no genetic information, or missing ancestry were excluded, as detailed in the methods. The metrics used as exclusion criteria were established before analysis, but some thresholds used (e.g., cutoffs for ancestry

analysis for strata) were evaluated during the QC process. All the above exclusions were made in accordance with the planned study protocol and are detailed in the methods section.

Replication

To replicate genome-wide significant SNPs, we used publicly available meta-GWAS summary statistics from an independent, non-overlapping contemporary meta-GWAS of the EHR-defined spinal pain "dorsalgia" phenotype by Bjornsdottir et al. 2022 (n=119,100 cases, n=909,847 controls). This phenotype is similar to the CBP-MVP phenotype but includes cases of neck and back pain; we refer to this subsequently as the "GWAS of spinal pain". The GWAS of spinal pain used for replication included samples from deCODE Genetics (Iceland), the Danish Blood Donor Study (DBDS), Copenhagen Hospital Biobank (CHB), the UK Biobank (United Kingdom), and FinnGen (Finland). For analyses in the replication sample, the threshold for statistical significance was determined using a Bonferroni correction, with the conventional threshold of nominal significance (p<0.05) divided by the number of SNPs studied in the replication sample. We also report those associations that reach nominal (p<0.05) in the replication sample. Genetic correlations (rg) between CBP-MVP and publicly available spinal pain-related summary statistics31 (including the GWAS of spinal pain by Bjornsdottir et al.) were calculated to allow comparison of these phenotypes.

In the multi-ancestry meta-analysis of CBP-MVP, 57 variants replicated at the nominal threshold of significance (p<0.05) among 85 independent lead variants which had available summary statistics in Bjornsdottir et al. 26 variants replicated at the Bonferroni-corrected threshold (p<0.05/85=0.000556). In the EUR stratum, 49 of the 68 independent variants replicated at the nominal threshold of significance and 23 variants replicated at the Bonferroni-corrected threshold (p<0.05/68=0.00074). In the AFR ancestry stratum, 1 of the 2 lead variants replicated at the nominal threshold of significance (rs140875296, p=0.046), where it was reported at MAF=0.01. rs140875296 is likely AFR-specific (MVP MAF=0.042, 1000 Genomes African MAF=0.015) as it is not present in the MVP EUR stratum at >1% MAF nor is it detected in the 1000 Genomes EUR (MAF=0). rs10119541 was present in the sample reported by Bjornsdottir et al (MAF=0.037) and does not replicate (p=0.76); while present in both the MVP EUR and HIS strata (MAF=0.03) but does not replicate (p=0.059, p=0.63 respectively). See Supplemental File 1 sheets "Bjornsdottir_replic_multi_ances", "Bjorn_nominal_rep_multi_ances", "Bjorn_replication_EUR_ancestry", "Bjorn_nom_replic_EUR_ancestry", "Bjorn_nom_replic_AFR_ancestry" for variant replication summary statistics.

Randomization

Randomization of experimental groups was not applicable to this study. Our study assesses the observed association between the natural randomization of genotype and the ascertained phenotype of CBP.

Blinding

Blinding was not applicable to this study.

Reporting for specific materials, systems and methods

We require information from authors about some types of materials, experimental systems and methods used in many studies. Here, indicate whether each material, system or method listed is relevant to your study. If you are not sure if a list item applies to your research, read the appropriate section before selecting a response.

Materials & experimental systems

n/a	Involved in the study
☒	☐ Antibodies
☒	☐ Eukaryotic cell lines
☒	☐ Palaeontology and archaeology
☒	☐ Animals and other organisms
☒	☐ Clinical data
☒	☐ Dual use research of concern
☒	☐ Plants

Methods

n/a	Involved in the study
☒	☐ ChIP-seq
☒	☐ Flow cytometry
☒	☐ MRI-based neuroimaging

Plants

Seed stocks

Report on the source of all seed stocks or other plant material used. If applicable, state the seed stock centre and catalogue number. If plant specimens were collected from the field, describe the collection location, date and sampling procedures.

Novel plant genotypes

Describe the methods by which all novel plant genotypes were produced. This includes those generated by transgenic approaches, gene editing, chemical/radiation-based mutagenesis and hybridization. For transgenic lines, describe the transformation method, the number of independent lines analyzed and the generation upon which experiments were performed. For gene-edited lines, describe the editor used, the endogenous sequence targeted for editing, the targeting guide RNA sequence (if applicable) and how the editor was applied.

Authentication

Describe any authentication procedures for each seed stock used or novel genotype generated. Describe any experiments used to assess the effect of a mutation and, where applicable, how potential secondary effects (e.g. second site T-DNA insertions, mosiacism, off-target gene editing) were examined.
